# Cognitive performance, burden and stress in aged caregivers of older adults with and without cognitive impairment

**DOI:** 10.1590/1980-5764-DN-2022-0073

**Published:** 2023-06-26

**Authors:** Larissa Corrêa, Ana Carolina Ottaviani, Allan Gustavo Bregola, Nathalia Alves de Oliveira, Sirlei Ricarte Bento, Sofia Cristina Iost Pavarini

**Affiliations:** 1 Universidade Federal de São Carlos, Programa de Pós-Graduação em Enfermagem, São Carlos SP, Brazil.; 2 Universidade Federal de São Carlos, Departamento de Gerontologia, São Carlos SP, Brazil.; 3 Central London Community Healthcare NHS Trust, London, England.

**Keywords:** Caregivers, Cognition, Aged, Stress, Psychological, Family Health, Cuidadores, Cognição, Idoso, Estresse Psicológico, Saúde da Família

## Abstract

There is an increasing number of aged people who provide care for other older people. Commonly existing burden and stress can change the forms of cognitive performance depending on the context of the aged caregivers.

**Objective:**

To compare the cognitive performance, burden and stress of aged caregivers of older adults with and without signs of cognitive impairment.

**Methods:**

A cross-sectional and quantitative study conducted with 205 aged caregivers of older adults with signs of cognitive impairment and 113 aged caregivers of older adults without signs of cognitive impairment treated in Primary Health Care. They were evaluated for sociodemographic characteristics, cognition, burden, and stress. Descriptive (Kolmogorov-Smirnov test) and comparative (Student's *t*-test and Pearson's χ² test) analyses were performed.

**Results:**

Aged caregivers of older adults with signs of cognitive impairment were older, had lower schooling levels, and a higher percentage of daily care hours compared to the aged caregivers of older adults without signs of cognitive impairment. Regarding cognitive performance, the means were lower for all domains. In addition, this same group had higher scores, with a statistically significant difference for perceived stress and burden.

**Conclusion:**

Aged caregivers of older adults with signs of cognitive impairment showed lower cognitive performance, as well as higher burden and stress levels. These findings guide the planning of interventions with aged caregivers in the Primary Health Care.

## RESUMO.

Nota-se um crescente número de idosos que prestam cuidados a outros idosos. A sobrecarga e o estresse comumente existentes podem afetar de formas diferentes o desempenho cognitivo, a depender do contexto desses idosos cuidadores.

**Objetivo:**

Comparar o desempenho cognitivo, a sobrecarga e o estresse de idosos cuidadores de idosos com e sem indícios de alterações cognitivas.

**Métodos:**

Estudo transversal e quantitativo realizado com 205 idosos cuidadores de idosos com indícios de alterações cognitivas e 113 idosos cuidadores de idosos sem indícios de alterações cognitivas atendidos na Atenção Primária à Saúde. Eles foram avaliados quanto às características sociodemográficas, cognição, sobrecarga e estresse. Análises descritivas (teste de Kolmogorov-Smirnov) e comparativas foram realizadas (teste *t* de Student e χ² de Pearson).

**Resultados:**

Os idosos cuidadores de idosos com indícios de alterações cognitivas eram mais velhos, com menor escolaridade e maior percentual de horas diárias de cuidado em comparação aos idosos cuidadores de idosos sem indícios de alterações cognitivas. Já quanto ao desempenho cognitivo, as médias foram inferiores para todos os domínios. Além disso, esse mesmo grupo apresentou maior pontuação, com diferença estatisticamente significante, para o estresse percebido e para a sobrecarga.

**Conclusão:**

Os cuidadores de idosos com indícios apresentaram menor desempenho cognitivo e maiores níveis de sobrecarga e estresse. Tais achados norteiam o planejamento de intervenções voltadas aos idosos cuidadores na Atenção Primaria à Saúde.

## INTRODUCTION

A progressive increase in the number of aged people providing care to more dependent older adults is noted, that is, aged individuals taking care of other older individuals^1–3^. This caregiver profile needs attention, as age is considered a predictor for the decline in cognitive functions^4^. Besides, caregiving requires involvement in tasks that are cognitively complex and may affect the caregivers’ mental and physical health^5^, resulting in negative effects on the quality of self-care and care for others^6^.

Several studies indicate that caregivers of older adults have worse performance in cognitive processing, executive function, attention, memory and visuospatial ability compared to non-caregivers^7,8^. A literature review study evidenced that informal caregivers are more likely to have low cognitive performance, and the longer they are exposed to care tasks, the greater their cognitive decline, even after the aged person they cared for has died^9^.

International studies such as the cross-sectional one carried out with middle-aged caregivers in France^10^ and the population-based study conducted in Germany with aged caregivers^6^ identified that informal caregivers’ cognition is influenced by the care they offer, especially females^6^. In this way, the high stress levels resulting from the daily care burden can put the caregivers’ cognitive health at risk and make memory failure complaints emerge^11^.

Caregivers’ burden was considered a multidimensional response associated with the demand for care that can threaten physical and psychological well-being, and generate emotional discomfort^12^. It can be exacerbated by the negative impact of care provision and by the particularities of each older adult, such as neuropsychiatric symptoms, in the caregivers’ life^13,14^, as well as stress^15^, depression^15,16^ care time^17^, the fact of living with the older adult, and social and leisure restriction^18^.

A systematic review showed that there is diverse evidence of the negative impact of care on informal caregivers’ mental and physical health. Effects were observed especially in women, married caregivers, and those providing intensive care^5^.

Nevertheless, the results about the impact of care on the caregivers’ cognitive performance are still controversial and there are few studies addressing different groups of aged caregivers. Thus, the study hypothesis is that aged caregivers of older adults with signs of cognitive impairment (CI) have worse cognitive performance, increased stress and burden compared to aged caregivers of older adults without signs of cognitive impairment (WCI).

## METHODS

This is a cross-sectional and quantitative study, developed with aged caregivers treated in Primary Health Care from a municipality in the inland of the state of São Paulo, Brazilian southeast region.

The inclusion criteria for the aged caregivers were age 60 years or older, being registered in a Family Health Unit (FHU), and being a caregiver of a dependent older adult in the same house. To be considered care recipients, the older adults should require assistance for at least one basic activity of daily living (BADL) evaluated by the Katz Index^19^, and/or instrumental activity of daily living (IADL) according to the Lawton and Brody Scale^20^. These instruments were also applied to the aged caregivers, who should be independent or have a score of dependence on a smaller number of activities compared to the assisted older adults.

The exclusion criteria were the following: both aged individuals being independent for the activities of daily living (ADL), aged caregivers who had a severe hearing or visual impairments that could compromise their ability to answer the questionnaire, and those who had communication difficulties that prevented understanding the questions. In addition, cases of death of one of the aged individuals in the house, address change, and whether the older adult was not found at home after three attempts on different days and times, were also excluded.

The sample was selected from a total of 594 homes listed by the FHU teams, where two or more aged people lived. All homes were visited, except: 69 because the subjects were not found at home after three attempts; 28 due to change of address; 26 due to death of one of the aged individuals; 84 for refusal to participate; and 36 aged individuals were evaluated as independent for BADL and IADL in the same household.

The result was 351 interviewed dyads of aged caregivers and assisted older adults; among them, 33 were excluded due to incomplete evaluations. Therefore, the final sample consisted of 318 aged caregivers, divided into two groups: CI group (n=205) and WCI group (n=113). For the group of care recipient older adults without signs of cognitive impairment (OWCI), it was necessary a score higher than or equal to 65 points on the Addenbrooke Cognitive Examination — Revised (ACE-R). For the group of assisted older adults with cognitive impairment (OCI), a score below 65 points established by the ACE-R cutoff value was required^21^.

The data were collected through previously scheduled home interviews by trained researchers. The interviews took place in a single session, lasting approximately one hour and thirty minutes, from April to November 2014. The project was approved by the Research Ethics Committee of the Federal University of São Carlos (CAAE N. 45904621.7.0000.5504). The participation was voluntary and all of them signed the Free and Informed Consent Form.

The data on sociodemographic characterization and care were collected through a questionnaire prepared by the researchers containing diverse information as: gender (female/male), age (years), schooling (years), marital status (married/not married), care time (years), daily care (hours), and dependence level of the assisted older adult through BADL — Katz Index^19^ and IADL — Lawton and Brody Scale^20^.

Cognition was assessed using the ACE-R score, which varies from 0 to 100 points and considers different domains: orientation and attention (18 points), memory (26 points), verbal fluency (14 points), language (26 points), and visuospatial skills (16 points)^22^. For data comparison, the total mean of ACE-R and that of the domains were considered.

Care-related burden was verified utilizing the Zarit Burden Inventory (ZBI). It assesses the perceived impact of the act of caring on the caregiver's physical and emotional health, social activities, and financial standing. The total score varies from 0 to 88, and the higher the score, the greater the intensity presented by the caregiver^23^. For the analysis of this population, the total score was considered.

The Perceived Stress Scale (PSS) was also used; its score can vary from 0 to 56, with higher values indicating higher levels of perceived stress^24^. For the current study, the total score was considered.

The data were typed and validated with double-blind entry in Excel 2010 and exported to the Statistical Package for the Social Sciences (SPSS) software, version 21.0 (IBM Inc., Chicago, IL, USA). Subgroups were created to enable a comparative analysis of the groups CI and WCI. Descriptive statistics were performed for simple and percentage frequencies for the categorical variables and, for the continuous variables, the mean and standard deviation (SD) were calculated. Data normality was confirmed by means of the Kolmogorov-Smirnov test. For the comparison between the groups, the Student's *t*-test and Pearson's χ^2^; test were employed, with p≤0.05 as significance level.

## RESULTS

Table 1 presents the comparative analysis of the sociodemographic characteristics, performance in the ADL, and total cognitive performance of the assisted older adults.

**Table 1:** Comparison of the sociodemographic data, cognitive performance and basic and instrumental activities of daily living of assisted older adults with signs of cognitive impairment and those without signs of cognitive impairment. São Carlos, 2014–2015.

Characteristics of the assisted older adults		Total (n=318)	OCI (n=205)	OWCI (n=113)	p-value
Age – mean (SD)		73.3 (±8.0)	75.3 (±8.7)	70.0 (±5.9)	0.001*
Gender – n (%)	Male	226 (71.1)	133 (64.9)	93 (82.3)	0.001†
	Female	92 (28.9)	72 (35.1)	20 (17.7)	
Schooling – mean (SD)		3.5 (±3.6)	2.2 (±2.0)	6.2 (±4.0)	0.000*
BADL – mean (SD)		5.3 (±1.4)	5.1 (±1.6)	5.7 (±0.69)	0.000*
IADL – mean (SD)		14.1 (±3.8)	12.8 (±3.7)	16.3 (±2.6)	0.000*
ACE-R – mean (SD)		53.5 (±22.0)	40.5 (±15.2)	77.2 (±8.9)	0.001*

Abbreviations: SD: standard deviation; BADL: basic activities of daily living (assessed by means of the Katz Index); IADL: instrumental activities of daily living (assessed by means of the Lawton and Brody Scale); ACE-R: Addenbrooke Cognitive Examination – Revised; OCI: older adults with cognitive impairment; OWCI: older adults without signs of cognitive impairment. Notes:

* Student's *t* test;

† Pearson’ chi-square test.

Table 2 exhibits the comparative analysis of the sociodemographic characteristics and the care context for the groups of aged caregivers of older adults (CI and WCI).

**Table 2:** Comparison between the sociodemographic and care context data of the caregivers of older adults with cognitive impairment and those without cognitive impairment. São Carlos, 2014–2015.

Variables		Total (n=318)	CI (n=205)	WCI (n=113)	p-value
Age – mean (SD)		69.6 (±7.0)	70.6 (±7.5)	67.9 (±5.7)	**0.001** *
Gender – n (%)	Female	246 (77.4)	150 (73.2)	96 (85.0)	**0.014** **†**
	Male	72 (22.6)	55 (26.8)	17 (15.0)	
Marital status – n (%)	Married	290 (91.2)	181 (88.3)	109 (96.5)	**0.014** **†**
	Not married	28 (8.8)	24 (11.7)	4 (3.5)	
Schooling – mean (SD)		3.7 (±3.3)	2.6 (±2.5)	5.5 (±3.7)	**0.001** *
	No formal schooling	58 (18.2)	54 (26.3)	4 (3.5)	
	1–4 years	200 (62.9)	134 (65.4)	66 (58.4)	
	≥5 years	60 (18.9)	17 (8.3)	43 (38.1)	
Care time, years – n (%)	<5 years	161 (50.6)	111 (54.1)	50 (44.2)	0.091†
	≥5 years	157 (49.4)	94 (45.9)	63 (55.8)	
Hours devoted to care – n (%)	<5 hours	169 (53.1)	99 (48.3)	70 (61.9)	**0.020** **†**
	≥5 hours	149 (46.9)	106 (51.7)	43 (38.1)	

Abbreviations: SD: standard deviation; CI: aged caregivers of older adults with signs of cognitive impairment; WCI: aged caregivers of older adults without cognitive impairment. Notes:

* Student's *t* test;

† Pearson's chi-square test. Bold font indicates p-valor ≤0.05.

Table 3 displays the comparison of cognitive performance, burden and stress between the groups of caregivers.

**Table 3:** Comparison between the cognitive performance, burden and stress of the caregivers of older adults with cognitive impairment and those without cognitive impairment. São Carlos, 2014–2015.

Variable		Total (n=318)	CI (n=205)	WCI (n=113)	p-value
Cognitive performance – mean (SD)	Total	63.3 (±18.4)	57.8 (±17.9)	73.3 (±15.0)	0.001
	Attention and orientation	13.6 (±2.9)	13.0 (±2.9)	14.9 (±2.5)	0.001
	Memory	14.8 (±6.2)	13.3 (±5.9)	17.7 (±5.7)	0.001
	Verbal fluency	5.8 (±2.8)	5.2 (±2.7)	7. 0 (±2.7)	0.001
	Language	18.5 (±5.6)	16.8 (±5.6)	21.5 (±4.3)	0.001
	Visuospatial skill	10.3 (±3.7)	9.2 (±3.6)	12.2 (±2.0)	0.001
Burden		17.2 (±14.1)	18.6(±14.0)	14.6 (±14.0)	0.018
Stress		17.8 (±9.8)	18.7 (±10.2)	16.2 (±8.8)	0.029

Abbreviations: SD: standard deviation; CI: aged caregivers of older adults cognitive impairment; WCI: aged caregivers of older adults without cognitive impairment. Note: Student's *t* test.

## DISCUSSION

Regarding the sociodemographic profile of the assisted older adults, there were statistically significant differences for age, gender, and schooling, with the older adults from the CI group being older, with lower schooling levels, and dependent for most of the basic and instrumental activities, as well as presenting lower cognitive performance. The main differences presented by these older adults from the CI group refer to aspects mentioned in the literature as predictors of lower cognitive performance, such as low schooling level^25,26^, older age^25^, and greater dependence for ADL^27^.

Considering the aged caregivers, they were mostly women, had low schooling levels, and lived with the older adults they cared for. There were differences between the groups referring to age, schooling and care hours, with the CI group being older, with lower schooling mean, and more time devoted to care. The sociodemographic characteristics of the aged caregivers are similar to those observed in the national and international studies^28,29^.

The present study compared cognitive performance and the emotional aspects (burden and stress) among aged caregivers of older adults with and without signs of cognitive impairment. The caregivers of the CI group presented lower cognitive performance according to the ACE-R total score, 21% lower than the WIC group, and with higher burden and stress levels. In addition to that, it is noted that the CI group presented cognitive impairment with a lower mean in all cognitive domains compared to the WCI group, with an emphasis on a lower mean in verbal fluency.

The lower cognitive performance in the CI group can be explained by the sociodemographic characteristics (age, schooling), by care-related aspects (increased burden and stress evidenced and less time available for self-care)^30,31^, and by care provision for more cognitively compromised older adults^32^.

Regarding emotional health, it can be related to care responsibility. Women commonly assume most of the responsibility for the care provided, accumulating multiple activities, when in fact it could be shared with other family members and other spheres (state and society)^33^.

As the dependence level of the assisted older adult increases, the caregiver's stress level increases proportionally^34^. In turn, the stress symptoms are burden predictors, as well as depression and anxiety symptoms, which can cause serious harms to mental health^35^, even compromising the caregivers’ cognition^36^.

The dependence level of the assisted older adults, their physical and mental health conditions, and their cognitive capacity, in addition to other aspects related to the caregivers’ characteristics such as age, time devoted to the care, and social support received, among others, generate feelings of burden^16,28,37^ which can compromise the aged caregivers’ quality of life^38^, impair their sleep^39^, and harm performance of the care activities^40^.

It is noted that all the symptoms presented by the caregivers add up to a self-harm cycle, including an impairment in quality of life, which can increase the chance of declining cognitive performance by up to 3.4 times^41^. The same happens in the cases of people with altered sleep; when impaired, it is potentially harmful to the individual's cognition, mainly impairing memory and processing speed^42^.

If the caregivers’ cognitive performance is impaired, the care activities for the older adults can be potentially compromised, such as preparing meals and handling medications, as well as identifying health-related problems. A study carried out in Niterói demonstrated that caregivers of older adults with dementia showed cognitive decline, especially in the memory domain^43^. Although the study was developed in the context of caring for older adults with dementia, it is noted that those data were similar to our study. Therefore, we can assume that the existence of cognitive changes in the older adults from the CI group generate specific demands for each type of care, and that these demands influence perceived stress and burden. In turn, these emotions exerted a negative influence on the caregivers’ cognitive state^44^.

The present study provides important information about cognitive performance, burden and stress in aged caregivers of older adults with and without signs of cognitive impairment. The possibility of a broad gerontological evaluation by professionals from FHUs is noted, in order to early identify the demands of these aged caregivers and, thus, develop interventions aimed both at health promotion and disease prevention with the objective of improving their health and quality of life. These results are expected to support the development of public policies for this population and future research studies in order to identify effective interventions.

It is concluded that the groups were sociodemographically similar: prevalence of women, married individuals, and incomplete elementary school. Regarding total cognition and its domains, the CI group presented lower means in cognitive performance compared to the WIC group. The domain with the lowest mean was “verbal fluency” and the one with the highest mean was “language”. The caregivers from the CI group obtained higher scores for burden and perceived stress levels compared to the caregivers of older adults from the WCI group.

This study has limitations for its cross-sectional nature; it is not possible to verify the cause-and-effect relationship of the variables and generalize the data due to the specific sample of aged caregivers. For future research studies, we suggest meeting such demands and considering other variables, such as coping, and social support and social context of the aged caregivers.
